# Bacterial Pathogens Causing Ventilator-Associated Pneumonia in Mechanically Ventilated Children: A Systematic Review and Meta-Analysis

**DOI:** 10.7759/cureus.114735

**Published:** 2026-08-18

**Authors:** Mohamed M Elabd, Ahmed M Omran

**Affiliations:** 1 Pediatric Critical Care Unit, King Fahad Medical City, Riyadh, SAU; 2 Pediatric Intensive Care Unit, King Fahad Medical City, Riyadh, SAU

**Keywords:** bacteria, klebsiella, meta-analysis, multidrug resistance, nicu, pediatric, picu, pseudomonas aeruginosa, ventilator-associated pneumonia

## Abstract

Background: Ventilator-associated pneumonia (VAP) remains a major healthcare-associated infection in pediatric and neonatal intensive care units (PICUs and NICUs). The microbiological etiology of pediatric VAP varies considerably across geographic regions, and a comprehensive global quantitative synthesis spanning both PICU and NICU settings using modern quality appraisal frameworks is lacking.

Objectives: To estimate the global pooled prevalence of individual bacterial pathogens causing VAP in mechanically ventilated children and to characterize the burden of multidrug-resistant (MDR) organisms.

Methods: Following PRISMA 2020 guidelines and a prospective International Prospective Register of Systematic Reviews (PROSPERO)-registered protocol, we searched MEDLINE/PubMed, Scopus, Cochrane CENTRAL, and Web of Science for studies published between January 2015 and December 2025. Studies reporting bacterial pathogens isolated from children (0-18 years) on invasive mechanical ventilation for >= 48 hours were eligible. Pooled prevalence proportions and 95% confidence intervals (CIs) were estimated using the DerSimonian-Laird random-effects model after logit transformation. Subgroup analyses were performed by data reporting type (patient-based versus isolate-based). The certainty of evidence was evaluated using the Grading of Recommendations Assessment, Development, and Evaluation (GRADE) approach.

Results: Eighteen studies encompassing 1,524 VAP episodes across 13 countries were included. Gram-negative bacteria predominated, with a pooled prevalence of 76.30% (95% CI: 65.55-84.49%; I² = 93.0%). Gram-positive bacteria accounted for 13.73% (95% CI: 8.85-20.69%; I² = 88.2%). The most frequent individual pathogens were *Pseudomonas aeruginosa *(19.7%; 95% CI: 14.3-26.5%; I² = 86.4%) and *Klebsiella *spp. (18.8%; 95% CI: 12.8-26.9%; I² = 89.5%), followed by *Acinetobacter *spp. (9.4%), *Staphylococcus aureus* (7.2%), *Escherichia coli *(5.9%), *Stenotrophomonas maltophilia *(5.2%), and *Enterobacter *spp. (3.6%). Subgroup analysis by data reporting type revealed a borderline significant difference for *P. aeruginosa *(patient-based 24.4% vs. isolate-based 12.8%; p = 0.050) and a significant difference for *S. aureus *(patient-based 5.4% vs. isolate-based 13.6%; p = 0.022). The pooled prevalence of MDR pathogens was 23.47% (95% CI: 14.88-34.97%; I² = 89.3%). According to the GRADE assessment, the certainty of evidence was low for most outcomes due to high statistical heterogeneity and study limitations.

Conclusions: Gram-negative bacilli, particularly *P. aeruginosa *and *Klebsiella *spp., overwhelmingly dominate the global microbiological landscape of pediatric VAP. The substantial prevalence of MDR organisms, coupled with low certainty of global evidence, underscores an urgent need for institution-specific empiric antibiotic protocols and robust antimicrobial stewardship in pediatric critical care units worldwide.

## Introduction and background

Ventilator-associated pneumonia (VAP) is among the most common healthcare-associated infections in pediatric and neonatal intensive care units (PICUs and NICUs) and remains a major cause of morbidity, prolonged mechanical ventilation, extended intensive care unit (ICU) stay, increased healthcare costs, and mortality. VAP is generally defined as pneumonia that develops after at least 48 hours of invasive mechanical ventilation or within 48 hours following extubation. Despite advances in critical care practices, VAP continues to represent a significant challenge in pediatric critical care worldwide [1]. Clinically, its diagnosis relies on a combination of new or progressive pulmonary infiltrates on chest radiography alongside systemic and respiratory signs, including fever, purulent endotracheal secretions, worsening gas exchange, and microbiological confirmation [2].

The epidemiology of pediatric VAP differs substantially from that observed in adults. Reported incidence rates vary considerably according to patient population, ICU setting, geographic region, and diagnostic criteria, ranging from fewer than two to more than 20 episodes per 1,000 ventilator-days. Neonates, infants, and immunocompromised children are particularly vulnerable because of their immature immune systems, prolonged exposure to invasive devices, and the complexity of their underlying medical conditions [2].

The microbiological profile of pediatric VAP is characterized by considerable variability across healthcare settings. Gram-negative organisms, including *Pseudomonas aeruginosa*, *Klebsiella *spp., *Acinetobacter *spp., and *Escherichia coli*, are frequently reported as the predominant pathogens, whereas Gram-positive organisms, such as *Staphylococcus aureus*,* *contribute to a smaller but clinically important proportion of infections. Accurate knowledge of the microbiological epidemiology of pediatric VAP is essential for selecting appropriate empiric antimicrobial therapy while minimizing unnecessary broad-spectrum antibiotic exposure and supporting antimicrobial stewardship programs [3].

The increasing global burden of multidrug-resistant (MDR) pathogens has further complicated the management of pediatric VAP. Carbapenem-resistant *Acinetobacter baumannii*, extended-spectrum β-lactamase (ESBL)-producing *Klebsiella *spp., and methicillin-resistant *S. aureus *(MRSA) have emerged as major healthcare-associated pathogens associated with limited therapeutic options and poorer clinical outcomes [4]. Consequently, understanding the distribution of bacterial pathogens and MDR organisms has become increasingly important for optimizing empiric antimicrobial therapy and strengthening infection prevention strategies.

Although numerous observational studies have investigated the bacterial etiology of pediatric VAP, the available evidence remains fragmented because of differences in study design, patient populations, microbiological diagnostic methods, and geographic settings [5]. A quantitative synthesis of these studies is therefore needed to provide robust pooled prevalence estimates and identify consistent microbiological patterns across diverse pediatric intensive care settings.

Accordingly, this systematic review and meta-analysis was conducted to quantitatively synthesize the available evidence on bacterial pathogens causing VAP in mechanically ventilated children, estimate pooled pathogen-specific prevalence, characterize the burden of multidrug-resistant organisms, and describe the overall microbiological distribution of pediatric VAP.

## Review

Methods

Protocol and Registration

This systematic review and meta-analysis was conducted, executed, and reported in strict accordance with the Preferred Reporting Items for Systematic Reviews and Meta-Analyses (PRISMA 2020) statement [6]. The detailed study protocol was prospectively registered in the International Prospective Register of Systematic Reviews (PROSPERO) under the registration number CRD420251270332.

Eligibility Criteria

Studies were eligible for inclusion if they fulfilled all of the following criteria:

Population: Enrolled pediatric patients from birth (neonates) up to 18 years of age receiving invasive mechanical ventilation for at least 48 hours before the clinical diagnosis of VAP.

Setting: Conducted within PICUs, NICUs, or mixed pediatric critical care settings.

Diagnosis: Diagnosed VAP using clearly predefined clinical, radiological, and/or microbiological criteria.

Outcomes: Reported identifiable, granular bacterial pathogen data with sufficient numerical information to allow the extraction of raw prevalence counts or proportions.

Studies were explicitly excluded if they enrolled adult patients exclusively or failed to provide pediatric-segregated data, lacked an objective or clear definition of VAP, evaluated patients receiving non-invasive ventilation only, reported pneumonia manifesting prior to the initiation of mechanical ventilation, contained fewer than 10 VAP cases (to mitigate small-study effects), were published in languages other than English, or did not report identifiable pathogen-specific microbiological data.

Information Sources and Search Strategy

A comprehensive, systematic literature search was performed across electronic databases - including MEDLINE/PubMed, Scopus, Cochrane Central Register of Controlled Trials (CENTRAL), and Web of Science - for peer-reviewed studies published between January 2015 and December 2025. The search architecture utilized combinations of Medical Subject Headings (MeSH) terms and highly sensitive free-text keywords optimized for "ventilator-associated pneumonia", "pediatric and neonatal populations", and "bacterial pathogens". To maximize yield, the reference lists of all eligible studies and relevant secondary reviews were manually screened to identify additional citations via backward and forward citation searching.

Study Selection and Data Extraction

All retrieved bibliographic records were imported into Rayyan Qatar Computing Research Institute (QCRI), Doha, Qatar, and duplicate citations were programmatically and manually removed before screening [7]. Two reviewers independently screened the titles and abstracts against the eligibility criteria, followed by a rigorous, independent full-text assessment of all potentially eligible articles. Disagreements at any stage were resolved through open discussion and consensus.

Data extraction was performed independently by two investigators using a pre-piloted, standardized spreadsheet. Extracted parameters comprised: study demographics (first author, publication year, country, study design, study period, and specific ICU setting), sample characteristics (total number of VAP episodes), diagnostic criteria, microbiological specimen types (e.g., endotracheal aspirates (ETAs), bronchoalveolar lavage (BAL)), raw distribution counts of individual bacterial pathogens, and specific prevalence metrics for MDR organisms. Discrepancies were cross-verified and resolved by reaching a consensus after re-examining the primary source data.

For the purposes of this meta-analysis, MDR status was accepted as classified by the authors of each primary study, since a single standardized international definition of MDR was not uniformly applied across all included studies. This reliance on primary authors' definitions represents a source of clinical indirectness, which is reflected in our Grading of Recommendations Assessment, Development, and Evaluation (GRADE) [8] certainty assessment for the MDR outcome.

Methodological Quality and Certainty Assessment

The methodological quality and risk of bias of the included observational studies were independently evaluated using the Joanna Briggs Institute (JBI) Critical Appraisal Checklist for Prevalence Studies [9]. Each domain was systematically scored as "Yes", "No", "Unclear", or "Not Applicable".

To determine the strength and quality of the generated evidence, the certainty of the evidence for each primary pathogen-specific outcome was evaluated using the GRADE framework adapted specifically for systematic reviews of prevalence or evaluation data [10]. Major outcomes were classified into high, moderate, low, or very low certainty based on five core domains: study limitations (risk of bias), inconsistency (statistical heterogeneity), indirectness, imprecision, and publication bias.

Statistical Analysis

Meta-analysis of proportions was performed utilizing the DerSimonian-Laird (DL) random-effects model, selected as it remains the most widely applied and validated estimator for random-effects meta-analysis of proportions following logit transformation, facilitating direct comparability with previous pediatric and adult VAP meta-analyses employing the same statistical framework. To stabilize variances and normalize the distribution of proportions, a logit transformation was applied to individual study proportions prior to pooling, with final estimates back-transformed to the natural scale for clinical interpretation. A standard continuity correction of 0.5 was applied to studies reporting zero-event cells. Pooled prevalence estimates were expressed alongside their corresponding 95% confidence intervals (CIs).

Given the expected geographical and institutional variation, 95% prediction intervals (PIs) were additionally computed for primary outcomes to characterize the expected range of prevalence in future, distinct clinical settings. Statistical heterogeneity was evaluated via Cochran's Q test (significance set at p < 0.10) and quantified using the I² statistic, where I² values of approximately 25%, 50%, and 75% represented low, moderate, and high heterogeneity, respectively.

To explore potential sources of clinical and methodological heterogeneity, subgroup analyses were performed based on (1) the data reporting type (patient-based versus isolate-based reporting metrics) and (2) ICU setting (PICU versus NICU) for primary outcomes (Gram-negative bacteria, Gram-positive bacteria, *Klebsiella *spp., and *P. aeruginosa*), with statistical differences between subgroups evaluated via the omnibus Q test for subgroup differences (Q_b) within a meta-regression framework. Studies with mixed pediatric/neonatal populations that could not be reliably attributed to a single ICU setting were excluded from the PICU versus NICU subgroup analysis. Sensitivity analyses were performed using a systematic leave-one-out approach to determine the mathematical stability of the pooled estimates. Potential publication bias was evaluated through visual inspection of funnel plots after logit transformation and quantified via Egger's regression asymmetry test, restricted to outcomes supported by at least 10 independent studies to prevent artifactual asymmetry. All statistical computations were conducted using R software (version 4.3.2; R Development Core Team, Vienna, Austria) utilizing the meta and metafor packages [11].

Results

Study Selection

The systematic literature search identified a total of 1,686 records across the selected electronic databases. After the removal of 606 duplicate records, 1,080 unique citations remained for title and abstract screening. Following the initial screening phase based on titles and abstracts, 926 records were excluded, leaving 154 potentially eligible articles that underwent rigorous full-text assessment.

Of these 154 full-text articles, 136 were excluded based on predefined eligibility criteria for the following specific reasons: pediatric VAP data were unavailable or inextricably mixed with adult datasets (n = 24), non-English language publications (n = 13), publication date outside the predefined historical study period (n = 7), absent or non-identifiable bacterial pathogen data (n = 31), duplicate or overlapping datasets (n = 8), adult-only study populations (n = 14), review articles, editorials, or expert commentaries (n = 17), and failure to meet predefined VAP diagnostic criteria or unclear diagnostic definitions (n = 22).

**Figure 1:**
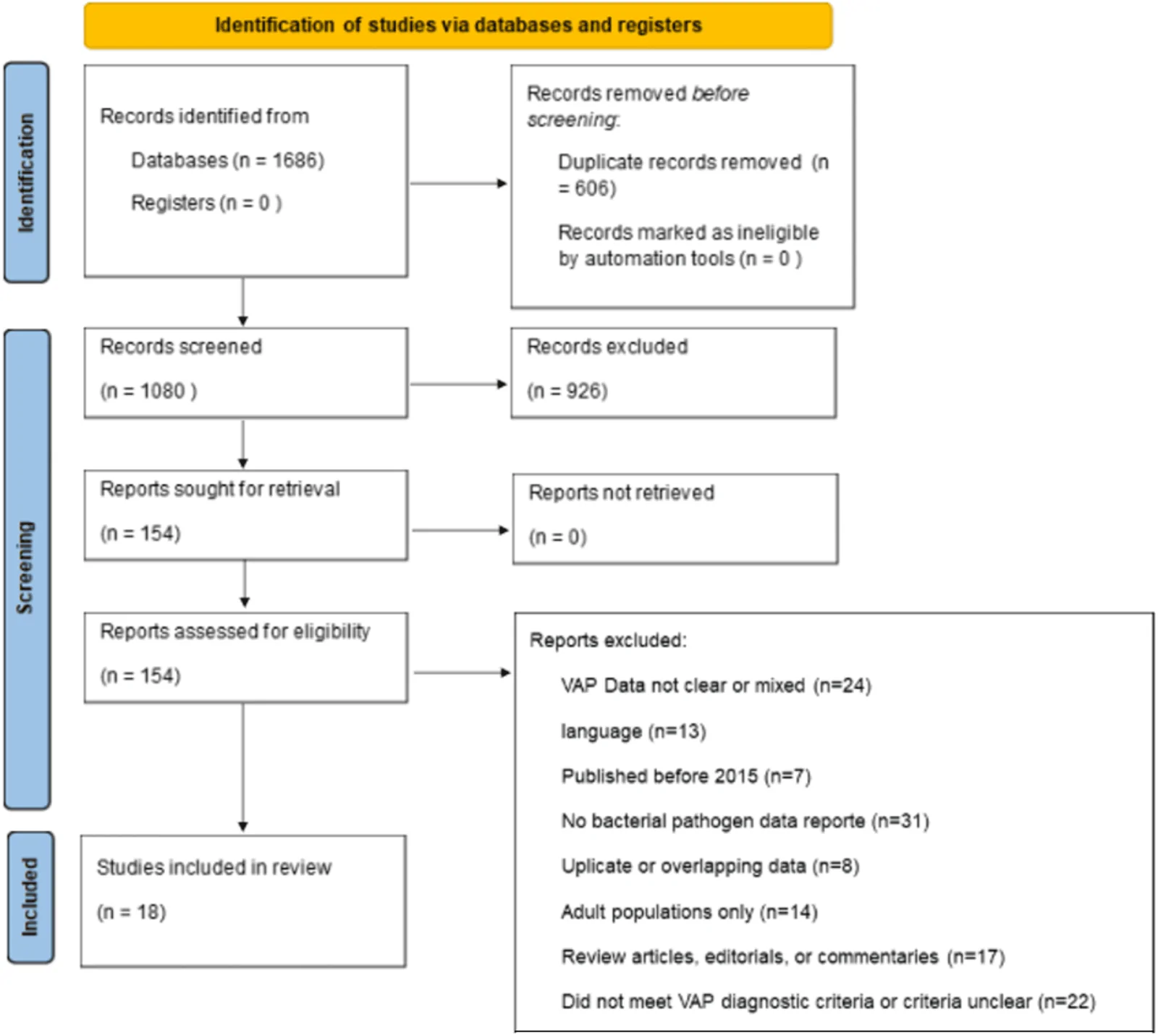
PRISMA 2020 flow diagram of the study selection The diagram illustrates the process of study identification, screening, eligibility assessment, and inclusion in the systematic review and meta-analysis.

Ultimately, 18 distinct studies met all structural eligibility criteria and were transitioned into the qualitative and quantitative synthesis (meta-analysis). The comprehensive multi-stage study selection process and attrition workflow are illustrated via the PRISMA 2020 flow diagram (Figure 1).

Characteristics of the Included Studies

A total of 18 studies comprising 1,524 pediatric VAP episodes were included in the meta-analysis. The studies were published between 2015 and 2024 and represented 13 countries across Asia, Africa, Europe, and North America. Study sample sizes ranged from 25 to 245 patients.

Regarding study design, nine studies were retrospective, eight were prospective, and one employed a case-control design. Ten studies were conducted in PICUs, six in NICUs, and two included mixed pediatric and neonatal critical care populations.

ETA was the most frequently used microbiological specimen for pathogen identification, although several studies used non-bronchoscopic bronchoalveolar lavage (NB-BAL), BAL, protected bronchial brushing (PBB), or other respiratory sampling techniques. VAP was diagnosed using established clinical and microbiological criteria, most commonly the Centers for Disease Control and Prevention (CDC) definitions [12], while several studies applied clinical/radiological criteria, modified Clinical Pulmonary Infection Score (CPIS), Neonatal Early-Onset Sepsis Calculator (NEO-KISS) criteria, or institution-specific diagnostic definitions. Detailed characteristics of the included studies are presented in Table 1.

**Table 1:** Characteristics of the studies included in the systematic review and meta-analysis Abbreviations: ETA, endotracheal aspirate; NB-BAL, non-bronchoscopic bronchoalveolar lavage; BAL, bronchoalveolar lavage; PBB, protected bronchial brush; TA/BAL, tracheal aspirate or bronchoalveolar lavage; TBA, tracheobronchial aspirate; PICU, pediatric intensive care unit; NICU, neonatal intensive care unit; CDC, Centers for Disease Control and Prevention; CPIS, Clinical Pulmonary Infection Score

First author (Year)	Country	ICU setting	Study design	Sample size	Study period	Respiratory specimen	VAP diagnostic criteria	Data type
Asanathong 2017 [13]	Thailand	Mixed	Retrospective	200	2009-2013	ETA	Clinical/Radiological	Patient-based
Atay 2021 [14]	Turkey	PICU	Retrospective	78	2011-2016	ETA	Clinical/Radiological	Patient-based
Azab 2015 [15]	Egypt	NICU	Prospective	73	2013-2014	NB-BAL	Clinical/Radiological	Patient-based
Bharathi 2022 [16]	India	PICU	Prospective	98	2016-2017	ETA	Clinical/Radiological	Patient-based
Bhattacharya 2023 [17]	India	PICU	Prospective	37	2017-2019	ETA	Clinical/Radiological	Patient-based
Chomton 2018 [18]	Canada	PICU	Retrospective	30	2013-2015	ETA	CDC 2015	Patient-based
El-Nawawy 2019 [19]	Egypt	PICU	Prospective	41	2015-2018	NB-BAL	CDC Criteria	Patient-based
Evren 2023 [20]	Turkey	PICU	Retrospective	42	2021-2022	ETA	CDC Criteria	Patient-based
Hamele 2015 [21]	USA	PICU	Retrospective	59	2009-2012	PBB	CDC Criteria	Isolate-based
Hernandez 2022 [22]	Spain	PICU	Retrospective	75	2015-2019	ETA	CDC Criteria	Patient-based
Liao 2020 [23]	Taiwan	NICU	Prospective	46	2017-2019	NB-BAL	CDC Criteria	Isolate-based
Manjhi 2018 [24]	India	PICU	Retrospective	135	2015-2017	ETA	CDC Criteria	Patient-based
Rangelova 2022 [25]	Bulgaria	NICU	Prospective	66	2017-2018	ETA	NEO-KISS/CDC	Isolate-based
Ratna 2024 [26]	Indonesia	NICU	Case-control	49	2020-2021	ETA	CDC Criteria	Isolate-based
van Wyk 2022 [27]	South Africa	PICU	Retrospective	31	2017-2018	TA/BAL	Modified CPIS	Isolate-based
Kushnareva 2019 [28]	Russia	Mixed	Retrospective	194	2010-2015	TBA/Oral Swab	VAP ≥48h	Patient-based
Scamardo 2020 [29]	Italy	NICU	Prospective	25	2013-2017	ETA	CDC Criteria	Isolate-based
Wang 2020 [30]	Taiwan	NICU	Prospective	245	2017-2020	ETA/NB-BAL	CDC Criteria	Isolate-based

Meta-Analysis Results

Gram classification: Gram-negative bacteria were the predominant causative organisms of pediatric VAP across all 18 included studies. The overall pooled prevalence of Gram-negative pathogens was 76.30% (95% CI: 65.55-84.49%; I² = 93.0%; p < 0.001). Stratification by data reporting type demonstrated a pooled prevalence of 82.48% (95% CI: 70.01-90.47%; I² = 92.5%; p < 0.001) among patient-based studies (k = 11, n = 1,003) and 65.32% (95% CI: 46.29-80.45%; I² = 91.6%; p < 0.001) among isolate-based studies (k = 7, n = 521).

**Figure 2:**
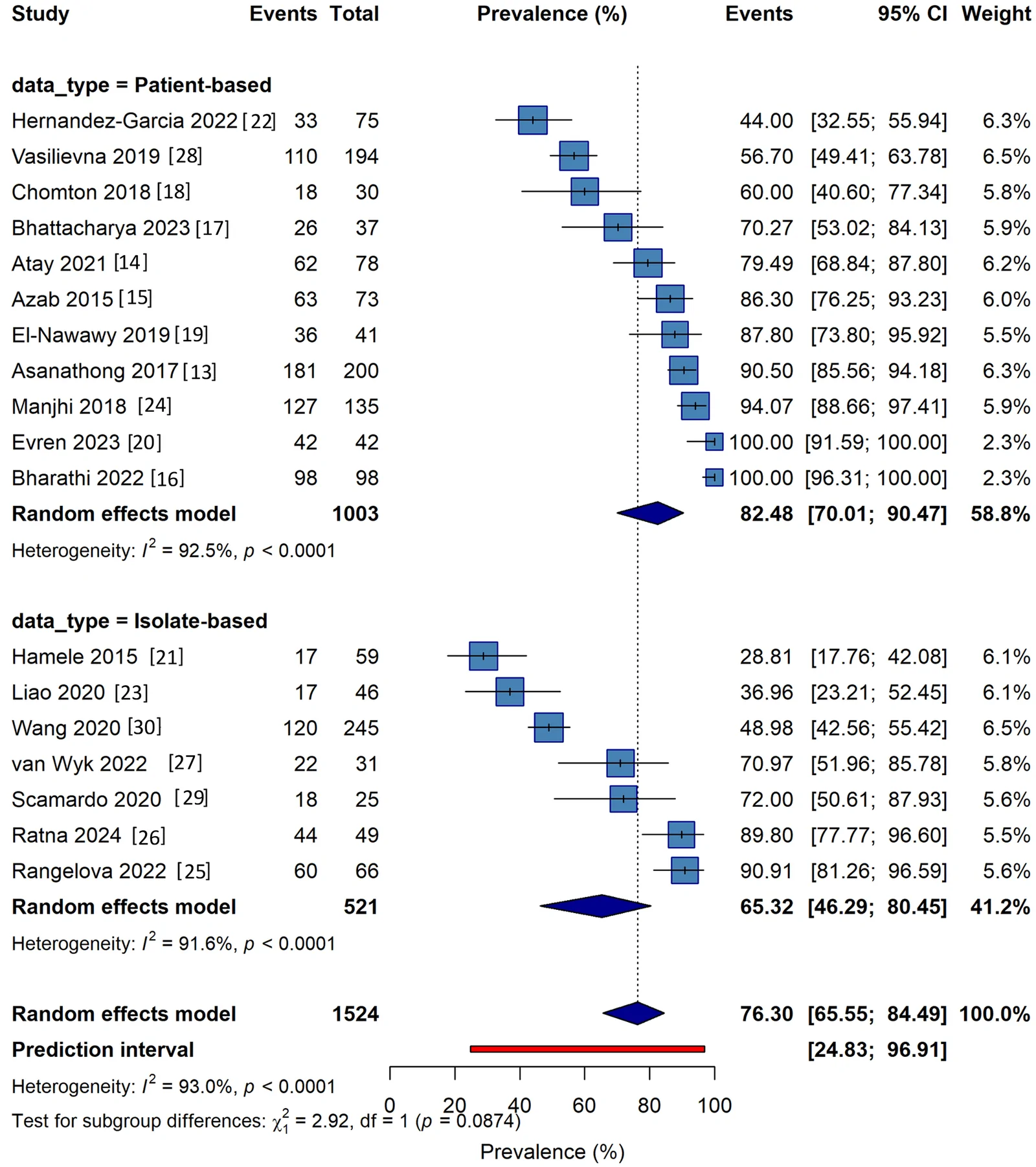
Forest plots of the pooled prevalence of Gram-negative bacteria causing ventilator-associated pneumonia in mechanically ventilated children The plot displays individual and overall prevalence estimates of Gram-negative pathogens, stratified into patient-based and isolate-based cohorts. Squares represent individual study estimates (size indicates statistical weight), and horizontal lines show 95% confidence intervals (CIs). The pooled prevalence estimates are shown at the bottom of each subgroup and overall analysis. Heterogeneity metrics (I² and Cochran's Q test) and the p-value for subgroup differences are provided at the base. Source: Refs [13-30]

The test for subgroup differences indicated that this variation between patient-based and isolate-based tracking metrics was not statistically significant (chi² = 2.92, df = 1, p = 0.0874). Strikingly, the 95% prediction interval ranged widely from 24.83% to 96.91%, structurally documenting the presence of extensive between-study variation and institutional heterogeneity in Gram-negative dominance across individual ICU settings (Figure 2).

Conversely, Gram-positive bacteria accounted for a significantly lower overarchingly pooled prevalence of 13.73% (95% CI: 8.85-20.69%; I² = 88.2%; p < 0.001). Subgroup evaluation showed that patient-based studies yielded a pooled Gram-positive prevalence of 10.25% (95% CI: 5.67-17.83%; I² = 86.0%; p < 0.001), while isolate-based studies demonstrated a higher pooled prevalence of 19.93% (95% CI: 9.67-36.66%; I² = 90.7%; p < 0.001).

**Figure 3:**
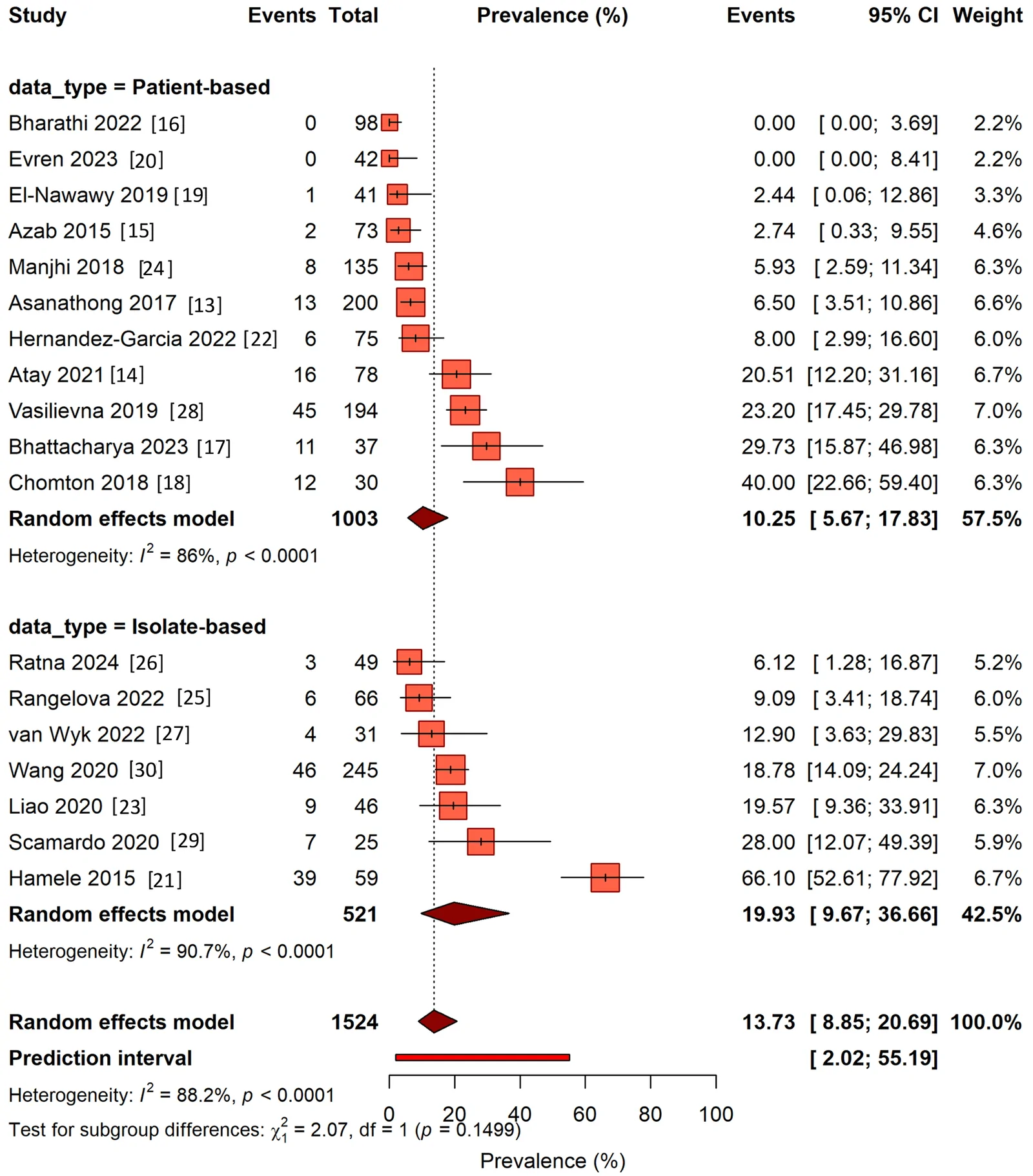
Forest plots of the pooled prevalence of Gram-positive bacteria causing ventilator-associated pneumonia in mechanically ventilated children The plot displays individual and pooled prevalence proportions of Gram-positive pathogens, categorized into patient-based and isolate-based tracking metrics. Squares indicate individual study effects (proportional to their weight), and horizontal whiskers indicate 95% confidence intervals (CIs). The extended horizontal line shows the 95% prediction interval (PI). Subgroup and overall statistical heterogeneity (I²) and the test for subgroup differences are presented at the bottom. Source: Refs [13-30]

The omnibus test confirmed that this difference between the statistical subgroups did not reach statistical significance (chi^2^ = 2.07, df = 1, p = 0.1499). The computed 95% prediction interval for Gram-positive pathogens spanned from 2.02% to 55.19%, structurally reflecting the prominent clinical and epidemiological diversity inherent across global critical care cohorts (Figure 3).

Individual pathogen prevalence: *P. aeruginosa *was identified as the single most prevalent individual bacterial pathogen across the synthesized cohorts, with an overall pooled prevalence of 19.7% (95% CI: 14.3-26.5%; I² = 86.4%; p < 0.001). Stratification by data reporting type revealed that patient-based studies yielded a higher pooled prevalence of 24.4% (95% CI: 17.6-32.8%; I² = 85.4%) compared to 12.8% (95% CI: 6.9-22.4%; I² = 77.0%) in isolate-based studies, demonstrating a borderline significant subgroup difference (chi² = 3.83, df = 1, p = 0.050). Of note, *P. aeruginosa *data were available for 17 studies, as Chomton et al. did not report individual metrics for this pathogen (k = 17) [18].

*Klebsiella *spp. represented the second most frequent pathogen, exhibiting an overall pooled prevalence of 18.8% (95% CI: 12.8-26.9%; I² = 89.5%; p < 0.001). No statistically significant variation was observed between patient-based studies (19.8%; 95% CI: 11.9-30.9%) and isolate-based studies (17.1%; 95% CI: 8.6-31.2%) (chi² = 0.12, df = 1, p = 0.724).

*Acinetobacter *spp. demonstrated a pooled prevalence of 9.4% (95% CI: 5.6-15.3%; I² = 86.9%; p < 0.001). Subgroup analysis showed no significant difference (chi² = 0.47, df = 1, p = 0.493) between patient-based (11.1%; 95% CI: 5.9-20.0%) and isolate-based estimates (8.4%; 95% CI: 5.0-13.7%).

*S. aureus *was the most dominant Gram-positive pathogen, accounting for an overall pooled prevalence of 7.2% (95% CI: 4.4-11.7%; I² = 81.4%; p < 0.001). A statistically significant subgroup difference was detected (chi² = 5.26, df = 1, p = 0.022), with isolate-based studies reporting a higher prevalence (13.6%; 95% CI: 7.2-24.2%) than patient-based studies (5.4%; 95% CI: 3.3-8.7%). This disparity likely reflects the compounding effect of counting multiple clinical isolates obtained from single patients over time in isolate-based tracking methodologies.

**Figure 4:**
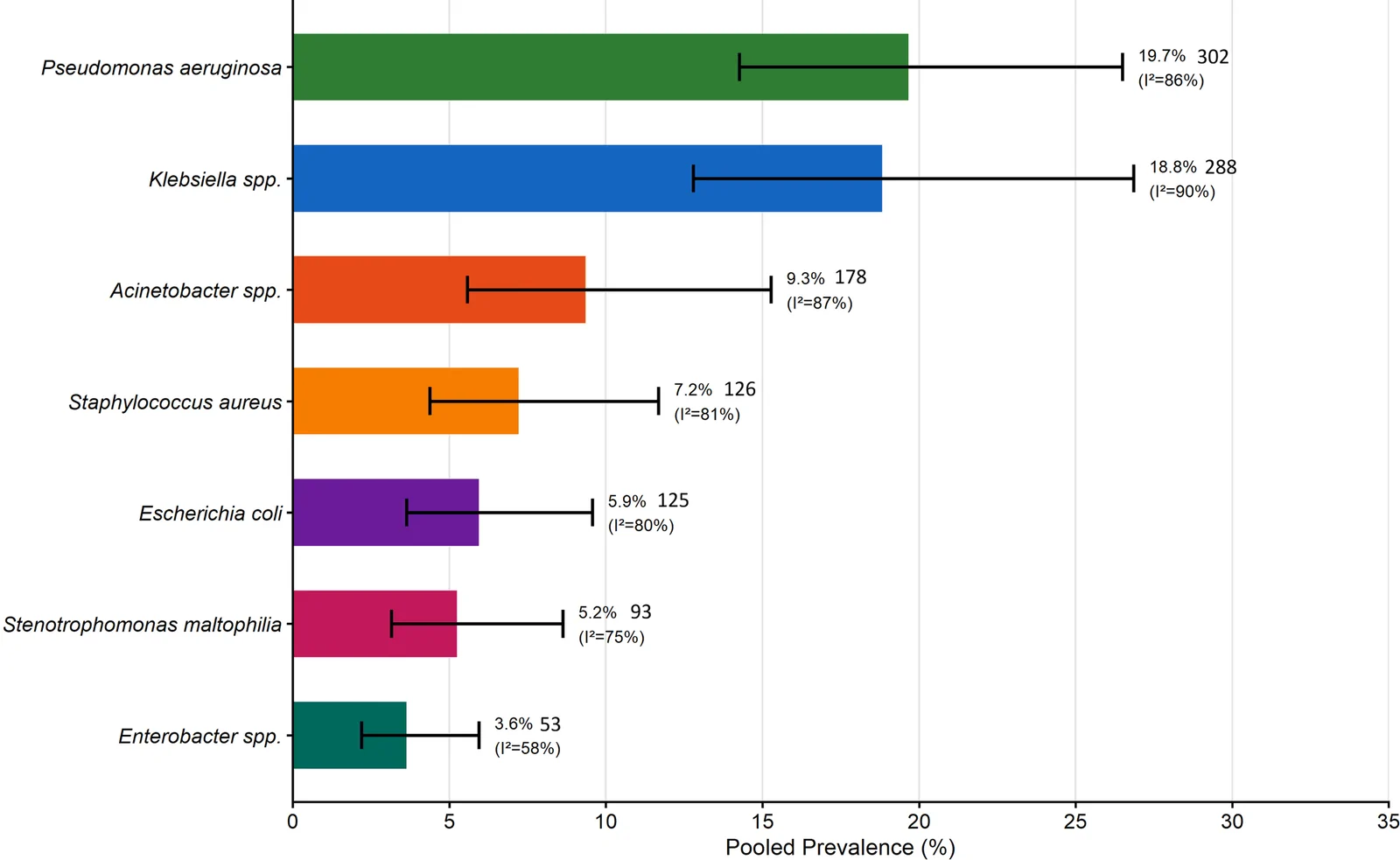
Summary bar chart of the overall pooled prevalence of individual bacterial pathogens causing ventilator-associated pneumonia (VAP) in children across 18 studies (total VAP episodes = 1,524) Bars represent the pooled prevalence percentages calculated via the random-effects model, ordered from highest to lowest frequency. Error bars indicate the corresponding 95% confidence intervals (CIs) for each specific pathogen.

*E. coli *exhibited a pooled prevalence of 5.9% (95% CI: 3.6-9.6%; I² = 79.6%; p < 0.001), with no significant subgroup variation (chi² = 0.05, df = 1, p = 0.825). Stenotrophomonas maltophilia accounted for 5.3% (95% CI: 3.2-8.6%; I² = 75.4%; p < 0.001), showing comparable rates between subgroups (chi² = 0.28, df = 1, p = 0.595). Finally, *Enterobacter *spp. demonstrated the lowest overall pooled prevalence at 3.6% (95% CI: 2.2-5.9%; I² = 57.9%; p = 0.001). However, a significant subgroup difference was observed (chi² = 5.78, df = 1, p = 0.016), with isolate-based studies yielding a higher prevalence (6.6%; 95% CI: 4.7-9.3%) compared to patient-based designs (2.0%; 95% CI: 0.8-5.0%). An overarching comparative summary of these pooled prevalence estimates is illustrated in Figure 4.

NICU versus PICU subgroup analysis: To further characterize potential differences in pathogen distribution by ICU setting, we performed subgroup analyses comparing PICU (k = 10, n = 626) versus NICU (k = 6, n = 504) for the four primary outcomes, excluding two studies with mixed pediatric/neonatal populations (Asanathong et al. [13] and Kushnareva et al. [28]) that could not be reliably attributed to a single setting.

The pooled prevalence of Gram-negative bacteria was comparable between PICU (78.4%; 95% CI: 60.9-89.4%) and NICU (74.6%; 95% CI: 53.0-88.4%) settings, with no significant subgroup difference (χ² = 0.11, df = 1, p = 0.745). Similarly, Gram-positive bacteria showed near-identical prevalence in PICU (13.1%; 95% CI: 5.6-27.7%) and NICU (13.1%; 95% CI: 7.7-21.3%) settings (χ² = 0.00, df = 1, p = 0.990).

For *Klebsiella *spp., the pooled prevalence was numerically higher in NICU settings (25.5%; 95% CI: 9.7-52.3%) compared with PICU settings (18.1%; 95% CI: 12.9-24.8%), consistent with our earlier observation that *Klebsiella *burden was concentrated in neonatal populations; however, this difference did not reach statistical significance (χ² = 0.48, df = 1, p = 0.486).

**Figure 5:**
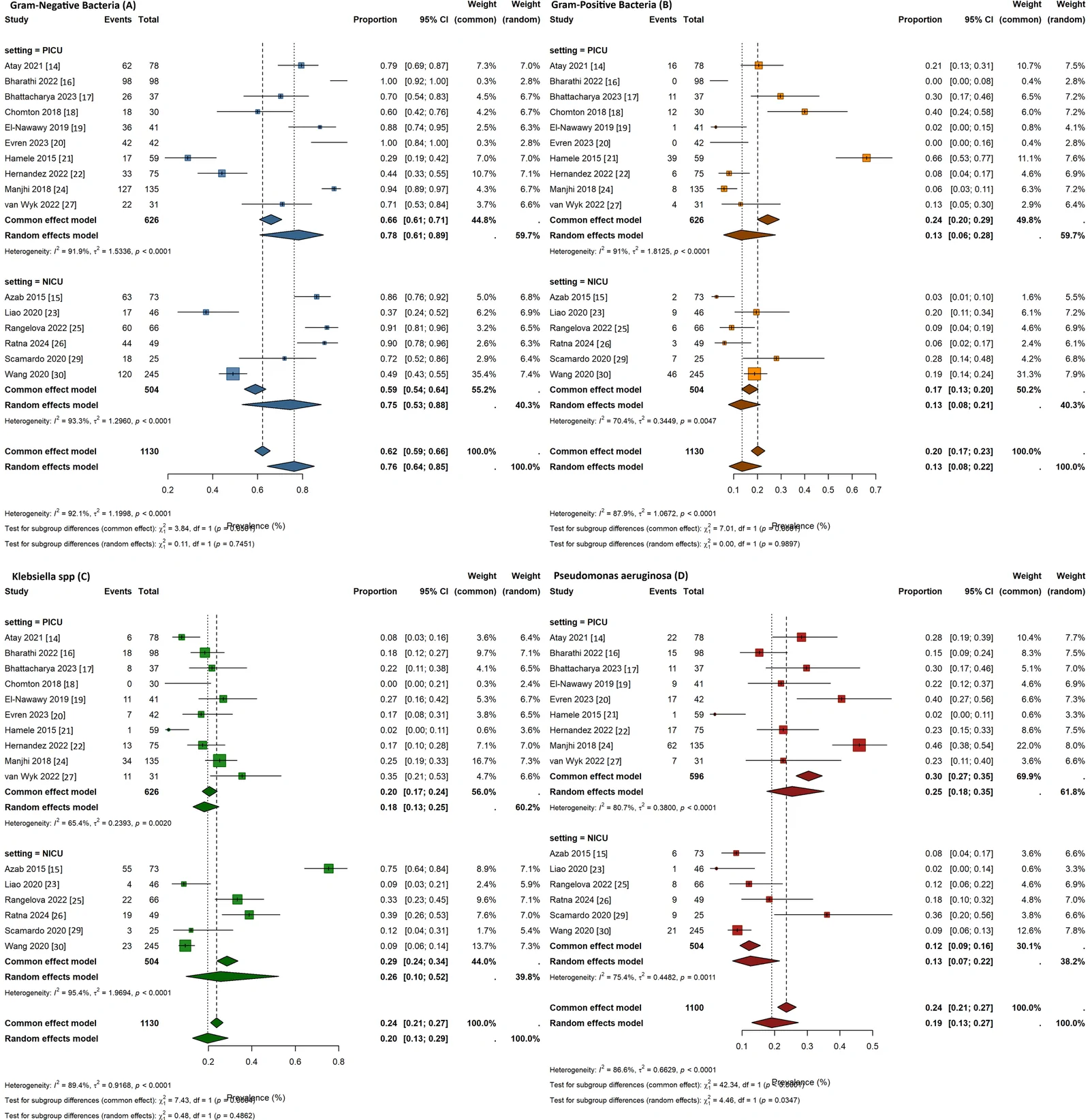
Forest plots of the pooled prevalence of key bacterial pathogens in pediatric ventilator-associated pneumonia, stratified by ICU setting (PICU versus NICU) Forest plots showing subgroup meta-analysis results for (A) Gram-negative bacteria, (B) Gram-positive bacteria, (C) *Klebsiella *spp., and (D) *Pseudomonas aeruginosa*, stratified by pediatric intensive care unit (PICU) versus neonatal intensive care unit (NICU) setting. Two studies with mixed pediatric/neonatal populations (Asanathong et al. [11], Kushnareva et al. [26]) were excluded from this analysis, as pathogen-specific data could not be reliably attributed to a single ICU setting. Squares represent individual study estimates, with square size proportional to study weight; horizontal lines indicate 95% confidence intervals. Diamonds represent pooled prevalence estimates using a random-effects model. Statistical heterogeneity was assessed using the I² statistic, and subgroup differences were tested using Cochran's Q test. A statistically significant difference between PICU and NICU settings was observed only for *P. aeruginosa* (p = 0.035), with higher prevalence in PICU settings. Source: Refs [13-30]

In contrast, *P. aeruginosa *showed a statistically significant subgroup difference (χ² = 4.46, df = 1, p = 0.035), with substantially higher prevalence in PICU settings (25.3%; 95% CI: 17.5-35.2%; k = 9) compared with NICU settings (12.6%; 95% CI: 7.0-21.5%; k = 6). Chomton et al. did not report *Pseudomonas*-specific data and were excluded from this analysis [18]. Forest plots for all four subgroup analyses are presented in Figure 5.

MDR pathogens: Among the 13 studies that systematically reported MDR data (with five studies failing to provide clear MDR definitions or tracking status and thus excluded from this specific analysis), the overall pooled prevalence of MDR pathogens causing pediatric VAP was 23.47% (95% CI: 14.88-34.97%; I² = 89.3%; p < 0.001).

Stratification by data reporting methodology demonstrated a pooled MDR prevalence of 23.51% (95% CI: 10.59-44.40%; I^2^ = 92.0%; k = 8) within patient-based cohorts, and a nearly identical pooled estimate of 23.42% (95% CI: 13.56-37.19%; I^2^ = 82.1%; k = 5) within isolate-based cohorts. The test for subgroup differences confirmed that this variation was not statistically significant (chi^2^ = 0.00, df = 1, p = 0.9922).

**Figure 6:**
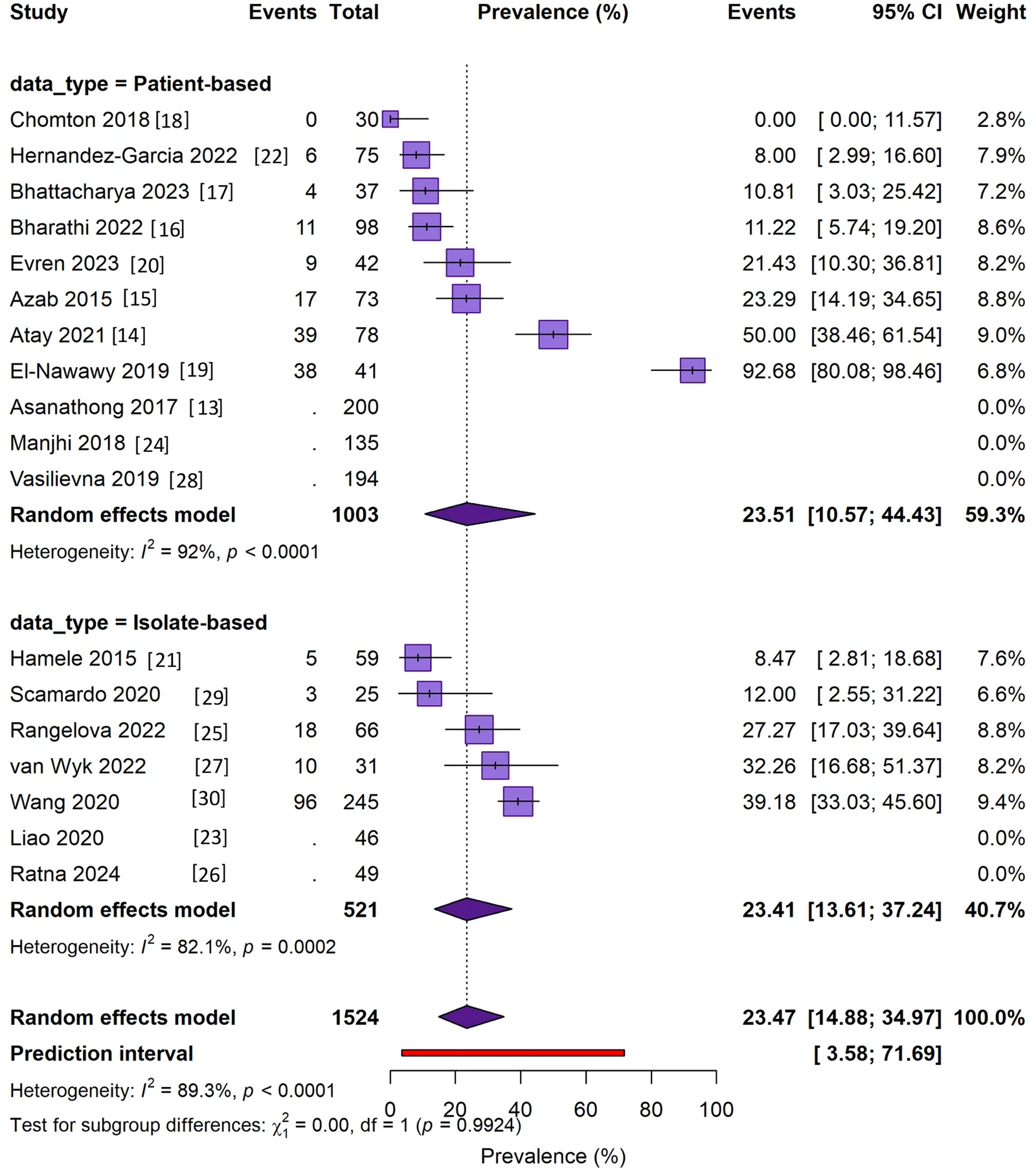
Forest plot of the pooled prevalence of multidrug-resistant (MDR) bacterial pathogens causing ventilator-associated pneumonia (VAP) in mechanically ventilated children Studies are stratified into patient-based and isolate-based cohorts. Squares represent individual study prevalence proportions (size denotes statistical weight), and horizontal lines indicate the 95% confidence intervals (CIs). overall pooled prevalence derived via the random-effects model, and the flanking horizontal lines represent the 95% prediction interval (PI). Source: Refs [13-30]

The computed 95% prediction interval spanned widely from 3.61% to 71.69%, structurally reflecting extreme institutional and geographical heterogeneity regarding the baseline MDR burden. Dynamically, the highest individual MDR prevalence was reported by El-Nawawy et al. at 92.7% [19], conducted in an Egyptian PICU environment, whereas the lowest baseline was documented by Chomton et al. at 0.0% in Canada [18]. Additionally, Wang et al. reported a substantial MDR prevalence of 39.2% within a Taiwanese NICU infrastructure [30]. Prominent MDR phenotypes systematically highlighted across the included literature comprised ESBL-producing *Klebsiella *spp., carbapenem-resistant *A. baumannii *(CRAB), and MRSA (Figure 6).

Sensitivity Analysis

Leave-one-out sensitivity analyses were systematically performed for all primary outcomes by sequentially excluding one individual study at a time and mathematically re-estimating the pooled prevalence from the remaining datasets. The results demonstrated robust and structurally stable pooled estimates across all evaluated microbiological outcomes.

For Gram-negative bacteria, the sequentially re-estimated pooled prevalence ranged narrowly from 74.10% (following the exclusion of Manjhi et al. [24]) to 78.50% (following the exclusion of Hamele et al. [21]), with all iterations remaining well within the 95% CI of the primary overarching estimate of 76.30% (95% CI: 65.55-84.49%). Similarly, the leave-one-out estimates for individual pathogens fluctuated minimally, ranging from 18.50% to 21.10% for *P. aeruginosa*, and from 17.20% to 20.10% for *Klebsiella *spp., fundamentally confirming that no single study exerted an undue or disproportionate mathematical influence on the core pathogen-specific estimates.

For MDR pathogens, the targeted exclusion of El-Nawawy et al. [19] - which documented the highest cohort-specific MDR prevalence of 92.7% - expectedly reduced the pooled estimate to 19.50%. Conversely, all other leave-one-out iterations for the MDR subset fluctuated tightly between 21.40% and 25.60%, remaining entirely consistent with the primary pooled baseline estimate of 23.47% (95% CI: 14.88-34.97%). Collectively, these sensitivity evaluations validate the mathematical robustness and reliability of all primary pooled prevalence proportions across the synthesized literature.

Publication Bias

To evaluate potential publication bias and small-study effects, funnel plots were visually inspected following logit transformation, and Egger's linear regression asymmetry test was formally applied for outcomes supported by at least 10 independent cohorts (k >= 10).

Egger's regression test indicated statistically significant funnel plot asymmetry for Gram-negative bacteria (t = 2.84, df = 16, p = 0.012) and *Acinetobacter *spp. (t = -4.59, df = 16, p < 0.001). Conversely, no statistically significant regression asymmetry was detected for *Klebsiella *spp. (t = -0.80, df = 16, p = 0.433) or *P. aeruginosa *(t = -1.91, df = 15, p = 0.076).

However, these findings should be interpreted with caution because funnel plot asymmetry may reflect both publication bias and substantial between-study heterogeneity. Given the substantial heterogeneity observed across the primary microbiological outcomes (I² = 75.4% to 93.0%), the observed funnel plot asymmetry is more likely to reflect genuine differences between studies, pronounced between-study variability in baseline pathogen distribution across diverse geographic regions, differing institutional antibiotic stewardship practices, and specialized ICU settings, rather than selective publication suppressions per se.

**Figure 7:**
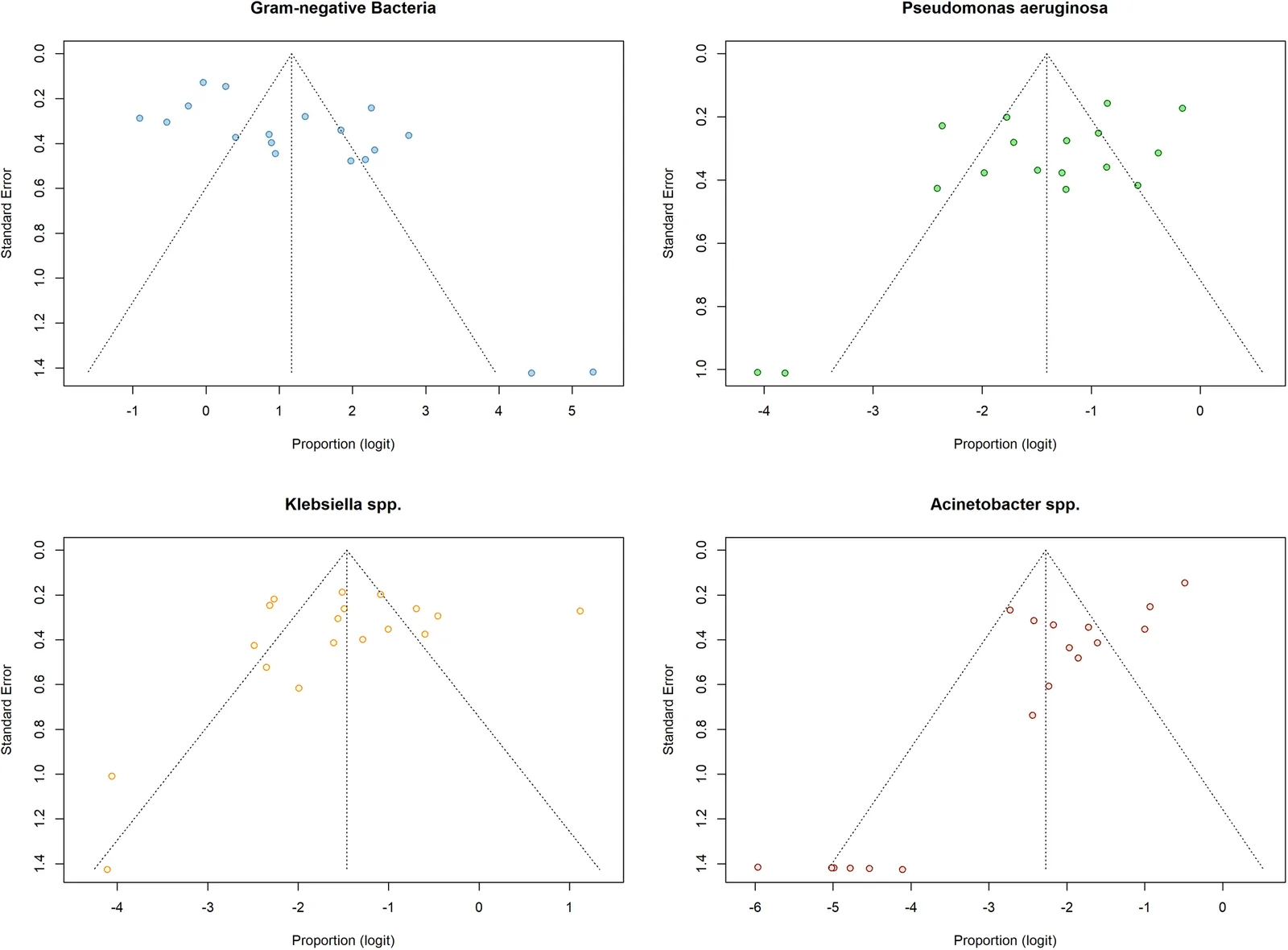
Logit-transformed funnel plots assessing publication bias for primary bacterial pathogen categories reported in at least 10 studies The plots include the studies reporting Gram-negative bacteria [13-30], *Pseudomonas aeruginosa *[13-17,19-30], *Klebsiella *spp. [13-30], and *Acinetobacter *spp. [13-30]. The vertical dotted line marks the overall pooled estimate, while the diagonal dashed lines define the pseudo-95% confidence intervals (funnel boundaries). Individual open circles represent study-specific prevalence estimates. The panels display standard error plotted against the logit-transformed prevalence proportions for Gram-negative bacteria, *P. aeruginosa*, *Klebsiella *spp., and *Acinetobacter *spp.

This epidemiological divergence aligns with established statistical limitations of asymmetry testing in prevalence meta-analyses characterized by substantial clinical heterogeneity. Nonetheless, because small-study effects cannot be definitively separated from selective reporting, the presence of localized publication bias cannot be entirely discounted, and this caveat should be considered during the clinical contextualization of the pooled estimates (Figure 7).

Methodological Quality and Risk of Bias Assessment

**Figure 8:**
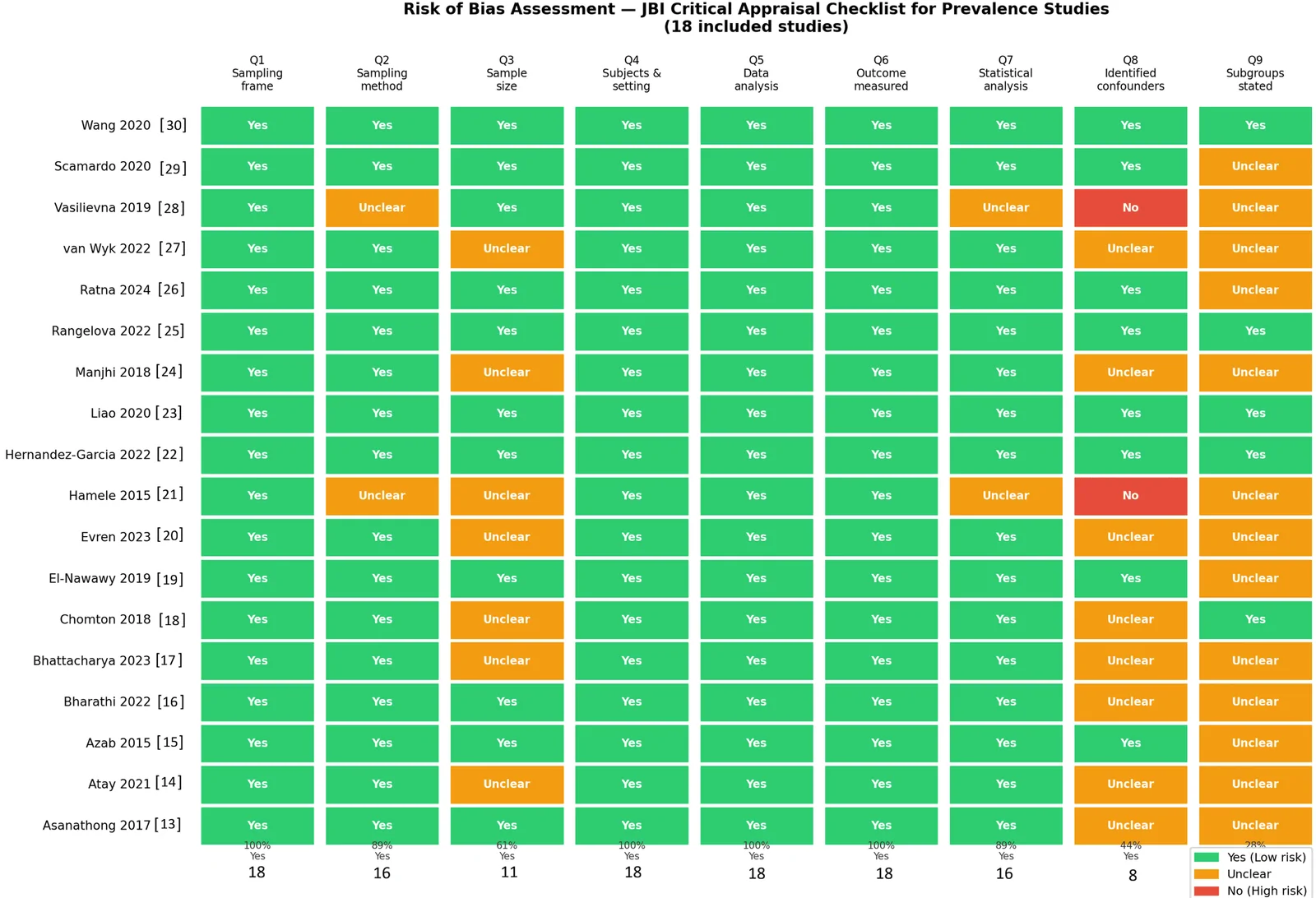
Methodological quality assessment of the included studies using the Joanna Briggs Institute (JBI) Critical Appraisal Checklist for Studies Reporting Prevalence Data Methodological quality assessment of the included studies using the Joanna Briggs Institute (JBI) Critical Appraisal Checklist for Studies Reporting Prevalence Data [10]. Green indicates criteria fulfilled ("Yes"), orange indicates "Unclear", and red indicates criteria not fulfilled ("No"). The percentages shown below each domain represent the proportion of studies meeting each criterion. Source: Refs [13-30]

Methodological quality assessment executed via the Joanna Briggs Institute (JBI) Critical Appraisal Checklist for Studies Reporting Prevalence Data [10] demonstrated that the overall methodological quality of the 18 included investigations was moderate to high (Figure 8).

A major strength of the included studies was the consistent use of clearly defined diagnostic criteria. All included studies were judged to have a low risk of bias ("Yes" rating) regarding the adequacy of the sampling frame (Q1), appropriateness of the study setting and target population demographics (Q4), data analysis coverage (Q5), and validation of diagnostic outcome measurements (Q6). Appropriate condition-specific sampling methods (Q2) and valid statistical analysis frameworks (Q7) were documented in 89% of the trials (16 out of 18 studies). However, an important methodological limitation was identified in sample size justification (Q3), where only 61% of the cohorts (11 out of 18 studies) provided explicit power calculations or sample size adequacy justifications, leading to "unclear" ratings across the remaining literature.

The main methodological limitations were inadequate adjustment for confounding and limited reporting of subgroup analyses. Specifically, adequate identification, adjustment, or reporting of potential confounding factors (Q8) was fulfilled by only 44% of the trials (eight out of 18 studies), with two investigations (Hamele et al. [21] and Kushnareva et al. [28]) exhibiting an explicit high risk of bias ("No" rating) due to the complete absence of confounding management. Furthermore, specific, well-defined subgroup analyses (Q9) were clearly stated and characterized in only 28% of the studies (five out of 18 studies), reflecting widespread generic reporting of baseline prevalence cohorts without intensive clinical stratification. Detailed, study-by-study quality evaluation scores across all nine operational JBI domains are visually structured in the risk of bias heatmap (Figure 8).

GRADE Summary of Findings

The certainty of evidence for all pooled prevalence estimates was systematically evaluated using the GRADE framework adapted specifically for prevalence studies (Table 2). Overall, the certainty of evidence was rated as "Low" for the majority of synthesized microbiological outcomes. This baseline rating primarily stems from the inherent observational cohort architecture of the included studies, moderate-to-serious methodological risks of bias, and substantial, unexplained between-study heterogeneity.

**Table 2:** GRADE summary of findings, derived from the included studies - bacterial pathogens causing pediatric VAP GRADE certainty ratings: ⊕⊕⊕⊕ High · ⊕⊕⊕◯ Moderate · ⊕⊕◯◯ Low · ⊕◯◯◯ Very low (Source: Ref [8]) Abbreviations: CI, confidence interval; PI, 95% prediction interval; I², inconsistency statistic; k, number of studies; MDR, multidrug-resistant; VAP, ventilator-associated pneumonia; PICU, pediatric intensive care unit; NICU, neonatal intensive care unit; JBI, Joanna Briggs Institute; ETA, endotracheal aspirate; BAL, bronchoalveolar lavage; CRAB, carbapenem-resistant *Acinetobacter baumannii*; ESBL, extended-spectrum β-lactamase; MRSA, methicillin-resistant *Staphylococcus aureus*. Source: Refs [13-30]

Outcome	Studies	Participants	Pooled prevalence (95% CI)	Certainty of evidence	Explanation
Gram-negative bacteria	18	1524	76.3% (65.6-84.5)	⊕⊕◯◯ Low	Downgraded for serious study limitations and very serious inconsistency (I²=93%).
Gram-positive bacteria	18	1524	13.7% (8.9-20.7)	⊕⊕◯◯ Low	Downgraded for serious study limitations and very serious inconsistency (I²=88%).
Pseudomonas aeruginosa	17	1494	19.7% (14.3-26.5)	⊕⊕◯◯ Low	Downgraded for serious study limitations and very serious inconsistency (I²=86%).
*Klebsiella *spp.	18	1524	18.8% (12.8-26.9)	⊕⊕◯◯ Low	Downgraded for serious study limitations and very serious inconsistency (I²=90%).
*Acinetobacter *spp.	18	1524	9.4% (5.6-15.3)	⊕⊕◯◯ Low	Downgraded for serious study limitations and very serious inconsistency (I²=87%).
Staphylococcus aureus	17	1494	7.2% (4.4-11.7)	⊕⊕◯◯ Low	Downgraded for serious study limitations and very serious inconsistency (I²=81%).
Escherichia coli	17	1494	5.9% (3.6-9.6)	⊕⊕◯◯ Low	Downgraded for serious study limitations and very serious inconsistency (I²=80%).
Stenotrophomonas maltophilia	18	1524	5.3% (3.2-8.6)	⊕⊕◯◯ Low	Downgraded for serious study limitations and very serious inconsistency (I²=75%).
*Enterobacter *spp.	18	1524	3.6% (2.2-5.9)	⊕⊕⊕◯ Moderate	Downgraded for serious inconsistency (I²=58%).
MDR pathogens	13	900*	23.5% (14.9-35.0)	⊕◯◯◯ Very low	Downgraded for study limitations, very serious inconsistency (I²=89%), and indirectness due to heterogeneous MDR definitions and incomplete reporting.

Characteristically, the certainty of evidence for *Enterobacter *spp. was uniquely rated as "Moderate," structurally reflecting a comparatively lower and acceptable level of statistical inconsistency (I² = 57.9%). In stark contrast, the certainty of evidence for MDR pathogens was downgraded to "Very low" due to a combination of serious risks of bias, profound statistical heterogeneity, and clinical indirectness arising from inconsistent or highly heterogeneous operational MDR definitions and incomplete resistance reporting across the historical studies (Table 2).

Discussion

To our knowledge, this study represents the first systematic review and meta-analysis to quantitatively synthesize the global bacterial etiology of VAP among mechanically ventilated pediatric patients across diverse healthcare settings. By pooling data from 18 high-quality studies involving 1,524 VAP episodes conducted in 13 countries spanning four continents, our findings provide a comprehensive, rigorous overview of the contemporary microbiological landscape of pediatric VAP.

Four main findings emerged from this meta-analysis. First, Gram-negative bacteria were the predominant pathogens, accounting for approximately three-quarters of all reported pathogens (76.30%), confirming that Gram-negative organisms remain the principal drivers of pediatric VAP worldwide. Second, *P. aeruginosa *(19.7%) and *Klebsiella *spp. (18.8%) were consistently identified as the leading causative pathogens, together representing nearly 40% of all reported bacterial isolates globally. Third, MDR organisms constituted almost one-quarter (23.47%) of reported pathogens, highlighting a critical and escalating challenge of antimicrobial resistance in critically ill children housed within intensive care infrastructures. Finally, subgroup analyses demonstrated that methodological differences in data reporting metrics (patient-based versus isolate-based tracking designs) significantly influenced prevalence estimates for selected pathogens, emphasizing an urgent need for standardized microbiological and surveillance reporting in future pediatric VAP research.

Comparison With the Previous Pediatric Literature

Direct comparison with previous pediatric evidence is limited because most published systematic reviews have historically evaluated the broader clinical epidemiology, incidence, risk factors, or prevention bundles of pediatric VAP rather than its specific microbiological profile, completely lacking quantitative pooling of individual bacterial pathogens.

In a neonatal-focused systematic review and meta-analysis restricted exclusively to NICU populations, Erfani et al. [22] reported a pooled Gram-negative prevalence of 72.0% among neonatal VAP isolates, which is closely comparable to our overall pooled estimate of 76.30% (95% CI: 65.55-84.49%) [31]. The same authors identified *Klebsiella *spp. as the leading Gram-negative organism among neonatal isolates, accounting for 41.0% of Gram-negative bacteria specifically. However, this figure is not directly comparable to our pooled *Klebsiella *estimate of 18.8% of all pathogens, since the mathematical denominators differ fundamentally. The Erfani et al. [31] estimate represents a proportion strictly confined to Gram-negative neonatal isolates within an NICU-restricted environment, whereas our metric represents a proportion among all isolated bacterial pathogens across mixed NICU/PICU cohorts spanning 13 countries.

In contrast to these prior restrictive or purely descriptive reviews, our study provides pooled prevalence estimates for individual bacterial pathogens across a broader, more heterogeneous global pediatric population, systematically evaluates the critical burden of MDR organisms, and explores the methodological impact of data reporting type, thereby substantially extending the current evidence base beyond generic descriptive epidemiology.

Heterogeneity of VAP Definitions/Pediatric Ventilator-Associated Events(PedVAE)

It is important to acknowledge that heterogeneity in VAP diagnostic definitions across the included studies (CDC clinical/microbiological criteria, modified CPIS, NEO-KISS, and institution-specific criteria) may have influenced case capture and, consequently, the observed microbiological profile [4]. Studies relying on broader clinical/radiological criteria may have captured a different case-mix than those applying stricter microbiological confirmation, potentially affecting pathogen distribution estimates. Furthermore, the increasing adoption of the PedVAE surveillance definition, which relies on objective respiratory support parameters rather than subjective clinical/radiological judgment, may shift future case ascertainment away from traditional VAP definitions [32]. This evolving diagnostic landscape underscores the importance of interpreting our pooled estimates within the context of the diagnostic criteria era in which the included studies were conducted and highlights the need for future studies to report pathogen distributions stratified by diagnostic definition.

Gram-Negative Predominance: Pediatric Versus Adult VAP

The marked predominance of Gram-negative bacteria observed in this meta-analysis (76.3%; 95% CI: 65.6-84.5%) is consistent with the well-established microbiology of VAP across intensive care settings regardless of patient age. Adult clinical practice guidelines and narrative reviews by Papazian et al., Kalil et al., and Torres et al. similarly describe Gram-negative bacilli as responsible for approximately 70-85% of VAP cases globally [33-35]. In a large systematic review and meta-analysis of adult VAP across Asia encompassing 31 studies, Bonell et al. [36] identified *A. baumannii *(26%), *P. aeruginosa *(22%), *Klebsiella pneumoniae *(14%), and *S. aureus *(14%) as the leading organisms.

The predominance of Gram-negative bacteria in both pediatric and adult populations suggests that ventilator-associated ecological pressures - including endotracheal tube biofilm formation, prolonged broad-spectrum antibiotic exposure, and ICU environmental colonization - rather than host age, constitute the dominant driver of Gram-negative predominance in VAP [33-35]. Although the overall Gram-negative dominance in our pediatric cohort closely parallels adult findings, the relative ranking of individual organisms differs notably: *A. baumannii *ranked first in the adult Asian cohort of Bonell et al. [36], at 26%, but only third in our pediatric analysis at 9.4%, while *P. aeruginosa *and *Klebsiella *spp. ranked higher in our pediatric population. Several factors may contribute to this divergence, including differences in ICU case-mix, regional antimicrobial resistance patterns, the proportion of NICU versus PICU studies, patient age-related immune differences, and the broader geographic representation of our review encompassing studies from Europe, Africa, and North America, in addition to Asia.

*P. aeruginosa*: It emerged as the single most prevalent individual pathogen in our analysis, with an overall pooled prevalence of 19.7% (95% CI: 14.3-26.5%; I² = 86.4%). This is closely comparable to the 22% reported by Bonell et al. [36] in adult Asian VAP, suggesting that *P. aeruginosa* maintains a consistently prominent role in VAP microbiology regardless of patient age. Its predominance is biologically plausible given its well-recognized virulence characteristics, including biofilm formation on endotracheal tubes, intrinsic resistance to multiple antimicrobial classes, environmental persistence within ICU settings, and the capacity to acquire additional resistance mechanisms during prolonged hospitalization.

A borderline significant subgroup difference was observed between patient-based studies (24.4%; 95% CI: 17.6-32.8%) and isolate-based studies (12.8%; 95% CI: 6.9-22.4%) (chi² = 3.83, df = 1, p = 0.050), suggesting that differences in reporting methods may influence prevalence estimates in *Pseudomonas *prevalence estimates. Regarding resistance burden, in a dedicated global meta-analysis of MDR *P. aeruginosa* in adult VAP encompassing 31 studies and 7,951 patients, Li et al. reported a pooled MDR prevalence of 33%, ranging from 19.7% in the United States to 87.5% in Iran [37]. We were unable to extract pathogen-specific MDR rates consistently across our pediatric dataset, precluding a direct pediatric versus adult resistance comparison for this organism; however, the magnitude of regional variability was reported by Li et al. [37]. mirrors the extreme heterogeneity we observed for overall MDR outcomes (I² = 89.3%), suggesting that local resistance epidemiology is the dominant determinant of MDR burden regardless of age group.

Notably, our subgroup analysis revealed a significantly higher prevalence of *P. aeruginosa *in PICU settings compared with NICU settings (25.3% vs. 12.6%, p = 0.035), which may reflect differences in patient age, underlying comorbidities, and duration of mechanical ventilation between these two populations.

*Klebsiella *spp.: It was the second most prevalent pathogen with a pooled prevalence of 18.8% (95% CI: 12.8-26.9%; I² = 89.5%) and appeared particularly concentrated in NICU-based studies. As discussed above, the 41.0% figure reported by Erfani et al. represents a proportion among Gram-negative neonatal isolates only and is not directly comparable to our all-pathogen denominator estimate [31]; no verified equivalent adult all-pathogen pooled *Klebsiella *prevalence was identified in the sources reviewed, and a numeric adult comparison for this organism is therefore not attempted.

The high prevalence of ESBL-producing *Klebsiella *reported in several included studies, notably Rangelova et al. [25], reporting 33.3% of all VAP isolates, and van Wyk et al. [27], reporting 32.3%, is particularly concerning, as ESBL production severely limits empiric treatment options and necessitates carbapenem use, which in turn risks driving further resistance [5]. These findings underscore the critical importance of active ESBL surveillance cultures, stringent contact precautions, and robust infection control programs in NICU settings, where *K. pneumoniae *has a well-documented propensity for nosocomial outbreaks.

*Acinetobacter *spp.: It accounted for 9.4% (95% CI: 5.6-15.3%; I² = 86.9%) of pediatric VAP episodes, substantially lower than the 26% reported by Bonell et al. in adult Asian VAP, representing one of the most striking pediatric-adult divergences in our dataset [36]. This difference likely reflects epidemiology rather than biology: the adult Asian cohort of Bonell et al. [36] was heavily weighted toward lower- and middle-income settings with a high endemic burden of CRAB, whereas our review drew from a broader geographic distribution, including European and North American centers with comparatively lower CRAB prevalence.

A lower pooled frequency should not be interpreted as lower clinical importance: in a large multicenter Italian adult cohort, Colaneri et al. found *Acinetobacter *in 9.7% of VAP isolates, closely comparable to our own estimate, with carbapenem resistance identified in 85.9% of those isolates, illustrating how resistance rather than raw prevalence rank drives the clinical impact of this organism [38]. A global meta-analysis by Mohd Sazlly Lim et al. further reported that MDR *A. baumannii *accounted for 79.9% (95% CI: 73.9-85.4%) of hospital-acquired *A. baumannii* VAP isolates worldwide [39], while a dedicated meta-regression by Hurley et al. demonstrated more than a fivefold variation in *Acinetobacter*-associated VAP incidence across seven world regions, unexplained by trauma case mix, publication year, or reporting practice [40]. Taken together, these findings suggest that carbapenem-sparing empiric strategies may be appropriate in lower-prevalence settings, while targeted CRAB coverage remains essential in high-endemicity regions, a geographic pattern consistent with the markedly higher *Acinetobacter *prevalence observed in Turkish and Egyptian studies within our own dataset.

S. aureus and Impact of Reporting Methodology

*S. aureus *was the most common Gram-positive pathogen with a pooled prevalence of 7.2% (95% CI: 4.4-11.7%; I² = 81.4%). This is substantially lower than the 14% reported by Bonell et al. [36] in adult Asian VAP and markedly lower than the 26.2% reported by Jackson et al. in a recent prospective 25-ICU, 11-country European network study, in which *S. aureus *was the most frequently identified VAP pathogen overall, with methicillin resistance in only 18.2% of *S. aureus *isolates [41]. The lower *S. aureus *prevalence in our pediatric cohort likely reflects genuine regional and age-related differences in VAP microbiology: pediatric cohorts with substantial NICU representation tend to be more Gram-negative-weighted, whereas the European adult ICU network of Jackson et al. reported a substantially larger *S. aureus* contribution.

An important methodological observation emerging from our analysis was the significant subgroup difference for *S. aureus *(chi² = 5.26, df = 1, p = 0.022), with isolate-based studies reporting a higher prevalence (13.6%; 95% CI: 7.2-24.2%) compared with patient-based studies (5.4%; 95% CI: 3.3-8.7%), likely reflecting the counting of multiple isolates per patient in isolate-based analyses. This finding has direct implications for the interpretation of *S. aureus *prevalence data across pediatric VAP studies and underscores the need for standardized reporting of microbiological denominators in future research.

Pathogens

The pooled MDR prevalence of 23.47% (95% CI: 14.88-34.97%; I² = 89.3%) represents a clinically significant finding and is best interpreted against pathogen-specific or PICU-wide surveillance comparators, since no directly equivalent pediatric VAP MDR meta-analysis was identified. In a dedicated global meta-analysis of MDR *P. aeruginosa *in adult VAP, Li et al. reported 33% MDR among *P. aeruginosa *VAP isolates specifically [37], while in a 50-PICU, 24-hour national point-prevalence study in Argentina, Cornistein et al. found that 20.6% of microbiologically confirmed PICU infections of any type were attributable to MDR organisms, with VAP the most frequent infection site [42]. These figures employ different denominators - pathogen-specific in Li et al. versus all-infection-type PICU point prevalence in Cornistein et al. [42] - and are not directly poolable with our estimate; however, together they suggest that our 23.47% pooled figure falls within a clinically plausible range.

The extreme 95% prediction interval (3.61-71.69%) and high heterogeneity (I² = 89.3%) reflect the dramatic geographic variability in MDR burden, ranging from 0.0% reported by Chomton et al. [18] (2018, Canada) to 92.7% reported by El-Nawawy et al. [19], which directly mirrors substantial variation in MDR prevalence across healthcare systems documented by Hurley et al. and Cornistein et al. [40-42]. Notable MDR organisms reported across our included studies included ESBL-producing *Klebsiella *spp., CRAB, and MRSA. Both *P. aeruginosa *and *Klebsiella *spp. possess well-recognized capacities to acquire resistance through extended-spectrum beta-lactamase production, AmpC beta-lactamases, carbapenemases, and efflux pump-mediated mechanisms, making pathogen-specific resistance surveillance an indispensable complement to pooled prevalence data for empiric therapy guidance [5,41].

Geographic Variation and Heterogeneity

Considerable geographic variation in pathogen distribution was observed across the included studies, reflecting the well-recognized influence of regional epidemiology on healthcare-associated infections. Studies conducted in South and Southeast Asia generally reported higher proportions of *P. aeruginosa*, *Klebsiella *spp., and *Acinetobacter *spp., whereas several European and North American studies demonstrated comparatively lower frequencies of these organisms and a relatively greater contribution of Gram-positive pathogens. This pattern is consistent with the strongest available multicenter evidence: Bonell et al. [36] demonstrated higher adult VAP incidence density in lower- and upper-middle-income Asian countries than in high-income countries, Jackson et al. [41] documented country-level VAP incidence varying from 7.6% to 29.6% within a single prospective European network, and Hurley et al. [40] demonstrated more than fivefold variation in *Acinetobacter*-associated VAP incidence across seven world regions, unexplained by case-mix or reporting practice.

The high statistical heterogeneity observed across all outcomes in our analysis (I² = 57.9% to 93.0%) parallels the extreme heterogeneity consistently reported in comparable regional meta-analyses and reflects genuine differences in microbiological epidemiology between healthcare systems, rather than representing a methodological weakness alone. These findings argue strongly against applying any pooled global prevalence estimate as a universal constant for empirical therapy selection and support our recommendation that empirical treatment and stewardship for pediatric VAP should be guided by local, institution-specific microbiological surveillance rather than extrapolation from pooled international estimates or adult antibiograms.

Clinical Implications

This review has several important clinical implications for the management of pediatric VAP. First, the overwhelming predominance of Gram-negative organisms, particularly *P. aeruginosa *and *Klebsiella *spp., supports maintaining adequate Gram-negative coverage when initiating empirical antimicrobial therapy in critically ill children with suspected VAP, consistent with recommendations by Papazian et al., Kalil et al., and Torres et al. [33-35]. Second, the substantial burden of MDR organisms - nearly one in four VAP episodes in our pooled analysis (23.47%) - highlights the importance of antimicrobial stewardship programs within pediatric ICUs, including early microbiological sampling, prompt de-escalation of antimicrobial therapy following culture results, and adherence to local prescribing guidelines. Crucially, the exceptionally wide 95% PIs observed across our primary outcomes - ranging from 24.8% to 96.9% for overall Gram-negative pathogens and from 3.6% to 71.7% for MDR organisms - underscore the profound inter-center and institutional heterogeneity across ICUs, mathematically demonstrating that global pooled prevalence rates cannot reliably predict the microbiological landscape of any individual unit. These pooled estimates should therefore complement, not replace, institution-specific microbiological surveillance and local antibiograms, as pathogen distribution and antimicrobial susceptibility patterns vary considerably across regions and healthcare settings.

Third, the methodological differences observed between patient-based and isolate-based reporting - significantly influenced prevalence estimates for *S. aureus* (p = 0.022) and *Enterobacter *spp. (p = 0.016) - underscore the need for standardized microbiological reporting in future pediatric VAP research, including clear specification of denominators, specimen collection methods, and pathogen-specific resistance profiles. 

Strengths and Limitations

This review has several strengths, including prospective PROSPERO registration, systematic multi-database searching, dual-reviewer screening and data extraction, use of logit transformation for proportion meta-analysis, prespecified subgroup analyses by microbiological reporting methodology, comprehensive leave-one-out sensitivity supporting the stability of the pooled estimates, and formal publication bias assessment using Egger's test.

Limitations include restriction to English-language publications; inability to perform formal subgroup analyses by geographic region or ICU type due to insufficient stratified data; variability in VAP diagnostic criteria and specimen collection methods across studies; the uncertain diagnostic validity of oral swab specimens used in one included study reported by Kushnareva et al. [28]; inconsistent MDR reporting in five of 18 studies; and the inability to disaggregate MDR prevalence by individual pathogen. All included studies were observational, and most were retrospective, which may have introduced residual confounding despite generally acceptable methodological quality. Future multicenter prospective studies employing standardized diagnostic criteria, harmonized microbiological reporting, and pathogen-specific antimicrobial susceptibility testing are essential to strengthen the evidence base and optimize the prevention and management of VAP in pediatric critical care units worldwide.

## Conclusions

This systematic review and meta-analysis provide a comprehensive quantitative synthesis of the bacterial etiology of pediatric VAP worldwide. Gram-negative bacteria were the predominant pathogens, with *P. aeruginosa *and *Klebsiella *spp. emerging as the leading pathogens across diverse geographic regions and intensive care settings. Furthermore, the substantial and alarming prevalence of MDR organisms highlights an escalating challenge of antimicrobial resistance in critically ill children, emphasizing the critical importance of continuous microbiological surveillance and robust antimicrobial stewardship programs within pediatric infrastructures.

Although pathogen distribution and resistance profiles varied considerably across the historical cohorts, the overarching findings demonstrate that empirical antimicrobial therapy should be tightly guided by local epidemiological data and institution-specific antibiograms, rather than relying solely on pooled international prevalence estimates. Additionally, the significant methodological variations observed between patient-based and isolate-based microbiological reporting underscore an urgent need for consensus-driven, standardized reporting practices to improve the comparability, transparency, and quality of future pediatric VAP research. Future large-scale, multicenter prospective studies using standardized diagnostic criteria, harmonized microbiological methods, and pathogen-specific antimicrobial susceptibility testing are needed to strengthen the current evidence base and optimize the management of pediatric VAP.
